# Factors related to turnover intention among staff of elderly caring social organizations in Anhui Province, China: a cross-sectional study

**DOI:** 10.1186/s12913-021-07284-5

**Published:** 2021-11-23

**Authors:** Xuefei Chen, Ling Tang, Liu Liu, Zhongliang Bai, Ren Chen

**Affiliations:** 1 School of Health Services Management, Anhui Medical University, Hefei, 230032 China; 2 The First Affiliated Hospital of Anhui University of Chinese Medicine, Hefei, 230031 China; 3 Affiliated Suzhou Hospital of Anhui Medical University, Suzhou, 234000 China

**Keywords:** Employee, Elderly caring social organizations, Turnover intention, Social support

## Abstract

**Objective:**

Turnover intention of employees in elderly caring social organizations has a significant impact on elderly care service delivery. This study investigated the associated factors of turnover intention among employees of elderly caring social organizations in Anhui Province, China.

**Methods:**

A total of 605 participants were selected using a multi-stage stratified random sampling method. A self-administered questionnaire was used to collect information on socio-demographic, social support, and turnover intention from the participants. The data was analyzed through descriptive statistical analysis, one-way variance analysis, Spearman correlation analysis, and multiple linear regression were used to analyze the factors related to turnover intention.

**Results:**

Results of our study showed that the total score of turnover intention, turnover intention I (possibility of quitting a current job),turnover intention II (motivation to find other jobs) and turnover intention III (obtaining the external possibility of work) were 8.84, 2.32, 2.38, and 4.14, respectively. Social support negatively correlated with turnover intention I and turnover intention II. However, it showed positive correlation with turnover intention III and total turnover intention scores; turnover intentionI (coefficient: − 0.082), turnover intention II (coefficient: − 0.071), turnover intention III (coefficient: 0.19), Total score of turnover intention (coefficient: 0.093). Ethnic group, age, education level, and job satisfaction were associated with turnover intention.

**Conclusion:**

Improvement of social support play an important role in reducing the turnover intention of employees in elderly caring social organizations. It is important to increase organizational commitment and strengthen psychological empowerment, combined with decreasing job burnout for stability.

## Background

China has experienced a rapid population aging in the recent years [1]. Issues that arise from this process, such as demographic transition, old-age pensions, life after retirement, and gerontotherapeutics, have increasingly drawn the attention of scholars [2, 3]. The aging of population has brought important challenges to the elderly care service provision. The traditional model of old-age care in the country is family old-age care. With the acceleration of modern life, young people with huge work pressure often feel powerless in caring for their parents. Therefore, people’s old-age care concepts are gradually changing. Many old people choose to enter elderly caring social organizations. Social organizations are also called non-governmental organizations, non-profit organizations, and third-party organizations (neither government organizations nor market organizations). Elderly caring social organizations are the main providers of social elderly services. The organizations are currently suffering from a shortage of human resources and irregular personnel [4]. Many people have a prejudice against the work of these employees and their hard work is hence not rewarded accordingly. This has led high turnover rate (exceeding 50%) among employees from elderly caring social organizations [5].

Turnover is an important value in human resources management and maintenance of workforce [6]. Turnover intention is believed to be a more revealing figure than the turnover rate since it is the antecedent of resignation, and has a better predictive ability [7]. Turnover intention is used to measure people’s intention to resign from their current job, look for another job and follow through on resigning [8]. Researchers describe turnover intention as the relative strength of desire of a person to voluntarily withdraw from an organization [9]. Intention is the reason, and turnover is the result. High turnover intention in deed predicted a greater likelihood of a person actually leaving a job [10]. High turnover intention among employees from elderly caring social organizations aggravated the shortage of human resources leading to decreased productivity and lowered elderly service quality [11]. Therefore studying the turnover intention of employees from elderly caring social organizations can stabilize the elderly care team. Several studies from developed countries have been conducted to investigate turnover intention and its influencing factors among employees from elderly caring social organizations [12]. However, such studies from developing countries are fewer.

The rapid aging of the Chinese population has led to high demand for social elderly care services. However, employees in these organizations have a higher turnover rate, associating with many negative influences on employees, organizations, and overall elderly caring service quality. Turnover intention is related to psychological distress and reduced work enthusiasm among the remaining staff members [13]. Recruitment and training of new employees incur costs and time consuming. Therefore, when trained workers leave an organization, it becomes a wasted investments [14]. In addition, a high turnover rate harms the quality, consistency and stability of social services provided by the organization [15].

Although studies have focused on employees in elderly caring social organizations, there is no adequate data on the relationship between the turnover intention among the employees and social support in China. Moreover, previous studies are mainly based on data from Western societies.

Social support is an organizational aspect that affects the retention of employees in social organizations. Available literature indicates that social support is an important factor in predicting turnover intention of employees [16]. Recently, the elderly caring social organizations have became an important part of social organization in China. However, the organizations are facing several challenges,for employees with low levels of peer support, the negative relationship between unsafe working conditions and low work commitment was stronger [17]. Low levels of social support was associated with low levels of workplace emotional commitment and high levels of turnover intention. Social support has a certain impact on turnover intention [18]. With a large number of elderly people requiring care in China, the employees have a heavy workload which increases their turnover intention.

To our knowledge, no studies to date have investigated the turnover intention and relevant determinants among staff of elderly caring social organizations in China. In light of Chinese ageing problem. Our study focused on employees in elderly caring social organizations in Anhui Province, China. It explored the status of employees turnover intention, the factors influencing turnover intention, and correlation of turnover intention with social support. The results of this study provides a reference information for stabilizing the employees team, improving quality of ageing services and reducing of turnover intention among employees of elderly caring social organizations for stability.

## Methods

### Studying setting

Anhui is located east of central China, and its economic development is at a medium level across the country. Anhui Province is the province that entered the aging society earlier among the 6 central provinces.

This province was selected for the following reasons: (1) Under the trend of the integration of elderly care in the Yangtze River Delta, many large-scale chain elderly care institutions have already forward-looking deployment in Anhui Province, and the government has also given great support and attention to this.(2) Considering feasibility, Anhui guaranteed the compliance of participants.(3) Anhui has a large number of employees in elderly caring social organizations.

### Study design and data collection

This study carried out a cross-sectional survey in the elderly caring social organizations of Anhui province, China. It was conducted between November 2019 and January 2020. The Biomedical Ethics Committee of Anhui Medical University gave an ethical approval for the study. We used a series of questionnaires to collect data and the employees answered the questionnaire themselves. If the respondent answered regularly to the questionnaires or filled in the questionnaires with incomplete content, it would be regarded as invalid and eliminated. Participants were enrolled using a multi-stage stratified cluster random sampling method. Six cities were selected from the sixteen prefecture-level cities in Anhui province (Fuyang, Huainan, Suzhou, Chizhou, Anqing, Lu′an). Determine all municipal districts, cluster sampling as the survey area, a total of 15 districts. In the district’s civil affairs bureau’s social organization account system that participates in elderly care services, a 50% percentage is selected, and there are no less than 20 A-type social organizations in each city. Because Type B social organizations have been established in a relatively short time, they are all subject to our investigation. A total of 252 elderly social organizations are selected. If the number of employees in the smaller organization is less than 3, all investigations will be conducted. If the number of employees in the larger organization is greater than 3, 2–3 people will be randomly selected..A total of 605 questionnaires were qualified for analysis.

### Measures

#### Measurement of turnover intention

Turnover intention was measured with a modified version of a turnover intention assessment scale,which consisted of 6 items. The six-item turnover intention scale (TIS-6), developed by Michaels et al. [19] and revised by Li et al. [20] for the Chinese population. A four-point Likert scale was used to rate the items, ranging from 1 (never, or strongly impossible) to 4 (always, or very possible). The total score of the six items was computed as the score for turnover intention, ranging from 6 to 24. A high score suggested a greater turnover intention (“≤ 15”comprised the“low”group and “> 15”comprised the“high”group). The scale includes a total of six items, divided into three dimensions: possibility of quitting a current job (turnover intention I, items 1 and 6), motivation to find other jobs (turnover intention II, items 2 and 3), and obtaining the external possibility of work (turnover intention III, items 4 and 5). The index value indicates the level of employee turnover intention (The ratio of actual score to theoretical highest score) (Table 2). The scale indicated a good internal consistency with a Cronbach’s alpha coefficient of 0.74.

#### Measurement of perceived social support

A 12-item self-report measure of social support was used to measure the perceived adequacy of social support for the participants [21, 22]. It used three sub-scales: family support, friend support, and other significant support. The respondents rated each item on a 7-point scale, from 1 (very strongly disagree) to 7 (very strongly agree). A total score was calculated by summing all the responses. Possible scores ranged from 12 to 84. A high score indicated a greater level of perceived social support. The Chinese version (MSPSS-C) has demonstrated high internal consistency with a Cronbach’s alpha equal to 0.89;therefore, its validity and reliability are confirmed [23].

#### Measurement of other variables

Demographic information and health-related variable data was collected, which include age (in years),sex (male, female), ethnic group (Han, ethnic minority), marital status (married/cohabited, single, never married, divorced, widowed), education (primary school and below, junior high school, college degree and above), have professional qualifications (yes, no), whether to accept business training (yes, no), working period (in years), satisfaction with current job (very dissatisfied, dissatisfied, fair, satisfied, very satisfied).

### Statistical analysis

Double entry and data validation was done in EpiData3.1. The collected data was analysed using SPSS 20.0 (IBM Corp, Armonk, NY, USA). Count data was expressed in composition ratio while measurement data was expressed in mean (M) ± standard deviation (SD).

For binary variables (gender, ethnic group, marital status, professional qualification certificate, Business training) univariate analysis was conducted using an independent sample *t*-test The age and working years of employees in elderly caring social organizations was changed into categorical variables. The age of employees was divided into four groups: 30 years and below, 31–50 years old, 51–70 years old and 71and above years. The working years was divided into four groups: less than 1 years, 1–5 years, 5–10 years and more than 1 years. For multiple categorical variables (age, education level, working years, satisfaction level) we performed a one-way ANOVA (analysis of variance) while Spearman correlation analysis was performed to determine the correlation between turnover intention and social support. The test level was set at *α* = 0.05.

Multiple linear regression models were done for multivariate analysis to set dummy variables for categorical variables. Set controls for each survey item were established to determine their association with other items using standard and non-standard coefficients, the test level *α* = 0.05.

## Results

### Demographic and characteristics of the study sample

We surveyed 605 people (190 males and 415 females). The average age of participants was 55.66 ± 11.56. The participants were largely Han (99.5%); Eighty four percent of the participants were married; their educational level was mainly primary school and below (48.6%). The working years were mostly 1–5 years (46.1%); The participants who had professional qualification certificates were 58.8% while those who did not receive any business training were 56%. Among them, 47.3% of employees were satisfied with their current job (Table 1).

**Table 1:** Descriptive results of participants characteristics (*N* = 605)

Variables	N = 605	Turnover intention	***t/F***	***P-value***
**Sex**				
Male	190(31.4)	8.54 ± 2.88	0.547	0.914
Female	415(68.6)	8.99 ± 3.37		
**Ethnic group**				
Han	602(99.5)	8.82 ± 3.19	19.454	< 0.001
Ethnic minority	3(0.5)	14.00 ± 6.92		
**Age (years)**				
≤ 30	28(4.6)	10.21 ± 3.39	1.893	0.021
31–50	129(21.3)	8.59 ± 2.61		
51–70	404(66.8)	9.04 ± 3.40		
≥ 71	44(7.3)	6.98 ± 2.29		
**Education level**				
Primary school and below	294(48.6)	8.42 ± 3.23	2.703	0.001
Junior school (High school)	245(40.5)	9.11 ± 3.23		
Junior college and above	66(10.9)	9.76 ± 2.97		
**Marital status**				
Married/cohabited	508(84)	8.89 ± 3.19	1.198	0.268
Single	97(16.03)	8.62 ± 3.19		
**Professional qualification certificate**				
Yes	249(41.2)	8.74 ± 3.11	0.543	0.916
No	356(58.8)	8.92 ± 3.31		
**Business training**				
Yes	266(44)	8.85 ± 3.00	0.897	0.568
No	339(56)	8.84 ± 3.40		
**Working years (years)**				
< 1	182(30.1)	8.93 ± 3.34	1.557	0.081
1–5	279(46.1)	8.99 ± 3.21		
5–10	91(15)	8.44 ± 3.03		
> 10	53(8.8)	8.57 ± 3.24		
**Satisfaction level**				
Very dissatisfied	1(0.2)	6 ± 0.00	3.174	< 0.001
Dissatisfied	5(0.8)	8.92 ± 5.89		
General	63(10.4)	9.65 ± 3.74		
Satisfaction	286(47.3)	8.79 ± 3.32		
Very satisfied	250(41.3)	8.59 ± 2.73		

### Scores of employees in elderly caring social organizations for turnover intention

The total turnover intention score was 8.84 ± 3.23 points, and the total average score was 4.42 ± 1.62. The turnover intention I, II and III scores were 2.32 ± 0.97, 2.38 ± 1.00, and 4.14 ± 2.38 respectively (Table 2).

**Table 2:** Total scores and scores of various dimensions turnover intentions among employees of elderly caring social organizations

Dimension	Theoretical highest score	Actual score	Index value
**Turnover intention I**	8	2.32 ± 0.97	0.29
**Turnover intentionII**	8	2.38 ± 1.00	0.30
**Turnover intention III**	8	4.14 ± 2.38	0.52
**Total score of turnover intention**	24	8.84 ± 3.23	0.37

*Note*: The index value is equal to the actual score divided by the theoretical maximum score. ≤0.25 means very low turnover intention; 0.25 ~ 0.5 means low turnover intention; 0.5 ~ 0.75 means higher turnover intention; ≥0.75 means very high turnover intention

### Correlation between social support and turnover intention of employees in elderly caring social organizations

Results of Spearman’s correlation analysis show that turnover intention I and II have a negative correlation with social support of the employees. Further, the turnover intention III and overall turnover intention were positively correlated with social support (Table 3).

**Table 3:** The relevance of turnover intention and social support

Project	Social support	
	***r***	***P-***value
**Turnover intention I**	− 0.082	0.013
**Turnover intentionII**	− 0.071	0.025
**Turnover intention III**	0.19	< 0.001
**Total score of turnover intention**	0.093	0.011

### Regression analysis

In linear regression analysis, score of intention to leave a job was the dependent variable, while the rest of scores were independent variables (Table 4). The results of multiple linear regression analysis showed that age, education level, ethnic group, satisfaction level, and social support were the influential factors of turnover intention (Table 4; *p* < 0.05). Employees whose education levels were junior college and above showed higher turnover intention than those whose education levels were primary school and below (Table 4; B = − 1.406). Differences in ethnic groups, age, satisfaction level and social support were also important factors with a direct correlation with the willingness of employees to leave (*p* < 0.05).

**Table 4:** Multiple linear regression model for turnover intention

Variables and groups	Unstandardized coefficients		Standardized coefficients	*t*	*p*-value	*95.0% CI*
	*β*	SE	*β*			
Constant	2.012	2.058		0.977	0.329	(−2.030,6.054)
Sex (male ^a^)						
Female	0.466	0.275	0.067	1.692	0.091	(−0.074,1.006)
Ethnic group (Han^a^)						
Ethnic minority	4.968	1.830	0.108	2.714	0.007	(1.370,8.560)
Age (years)	−0.0.498	0.202	−0.100	−2.464	0.014	(−0.900,-0.100)
Marital status (Married/cohabited ^a^)						
Single	0.292	0.355	0.033	0.823	0.411	(−0.406,0.991)
Professional qualification certificate	0.504	0.318	0.077	1.588	0.113	(−0.120,1.130)
Business training	−0.068	0.303	−0.011	−0.225	0.822	(−0.660,0.530)
Working years (years)	−0.013	0.150	−0.004	−0.087	0.930	(−0.310,0.280)
Education level (Junior college and above^a^)						
Primary school and below	−1.406	0.509	−0.218	−2.765	0.006	(−2.410,-0.410)
Junior school (High school)	−0.584	0.483	−0.089	−1.209	0.227	(−1.530,0.370)
Satisfaction level (very satisfied^a^)						
Satisfaction	0.436	0.275	0.068	1.587	0.113	(−0.104,0.977)
General	1.371	0.450	0.130	3.051	0.002	(0.489,2.254)
Dissatisfied	6.625	1.457	0.186	4.546	0.000	(3.763,9.487)
Very dissatisfied	−3.465	3.142	−0.044	−1.103	0.271	(−9.635,2.705)
Social support	0.025	0.010	0.100	2.435	0.015	(0.010,0.040)

^a^Constant

^*^p < 0.05

## Discussion

The turnover rate of employees working in elderly nursing homes for the aged in China is very high because it is not economically attractive [24]. Turnover intention can predict the actual resignation behavior of employees [25]. This study investigated the turnover intention and its relevant determinants among employees of elderly caring social organizations in Anhui, China. Overall, the study showed that the total turnover intention of 605 employees of the organizations was 8.84 ± 3.23. The index value is used to determine the level of turnover intention. The ratio was shown to be between 0.25 and 0.5. This ratio indicates that the employees of elderly caring social organizations in Anhui Province have low turnover intention, which is inconsistent with other previous studies [26].

That most employees are not willing to leave their current job, could be related to the vigorous promotion of elderly care services in China. In 2013, China included the requirement of “prioritize the development of social elderly services” in the “Twelfth Five-Year Plan” to encourage promotion of elderly care services. The government has also introduced various policies and increased capital investment for the development of the elderly care industry. That the score of turnover intention III (the probability of obtaining a new job) was the highest shows that employees of elderly social organizations are more likely to get other jobs. The employees have mastered work skills and effective communication methods during their working period hence are more likely to quit and find other organizations that need experienced employees. However, the scores of turnover intention I and turnover intention II are similar, indicating that employees of elderly caring social organizations are less likely to quit their jobs and have less motivation to find other jobs. The unwillingness to leave the current job may be related to the fact that the employees often establish a deep feeling of reluctance to leave.

The results of this survey indicates that there is significant difference in turnover intentions among employees of different ages in elderly caring social organizations of in Anhui Province, China. Young people (30 years and below) have the highest scores, while scores of older people (over 71) are relatively low. This finding is inconsistent with a previous study by whom [27], that showed young employees were more likely to leave their current jobs in China. Could be because old employees were more satisfied with their current the job situation. Employees in the age group of 51 to 70 years could have chose to remain in this stable job to continue supporting their children and grandchildren. The high turnover intention among young employees (30 years and below) could be due to the many choices they still have in their future career development. The young employees also have expectations for work organization, salary is and the job status different from the actual situations they find in the organizations. Furthermore, psychological imbalance of the young employees may have prompted them to to leave.

Employees from the minority ethnic groups showed a higher turnover intention than the rest of the groups. This could be due to presence of fewer employees of their ethnic groups in elderly caring social organizations. They could have faced challenges in their relationship and communication with colleagues, and other persons in the organization.

This study found that the likelihood of turnover intention was greater in employees with education level of junior college and above than those with level of primary school and below [28]. One possible explanation for this finding could be due to the late start of the development of elderly caring social organizations in the country. There is also immature career promotion and development of employees in the social organizations. Further, the managers of these institutions have not designed for careers progression of their employees, which disappoints the highly educated ones. Educated employees lack a platform to display their expertise, and are equally engaged in similar job with lowly qualified colleagues. They lack the sense of accomplishment they deserve and leave in search of better development prospects. Employees with low academic qualifications showed low turnover intention is low due to a job choices.

Job satisfaction was found to be the most important factor of employees’ turnover intention in China. It reduces organizational costs by lowering employee turnover, absenteeism, and thefts at work [29]. Turnover intention can be reduced by increasing job satisfaction because job satisfaction has a negative correlation with the intention to leave job [30]. Consistent with previous studies, various managerial interventions are being discussed for improvement of job satisfaction in China [31]. The government and organization managers should increase the salaries of employees, provide them with promotion opportunities and security insurance, such as health insurance and pension insurance.

The negative correlation found in both turnover intention I and turnover intention II with social support, shows that high social support, lows the possibility of quitting and lower motivation to search for other jobs. The social support among the employees mainly comes from the relationship between colleagues, leaders and the elderly persons in the caring social organization. Generally, employees have a short working life, their work is difficult, and lack proper communication among themselves such that when they encounter difficulties they can not get assistance. As service targets, the elderly also need to cultivate and inculcate close relationships with caregivers in order to reduce their tendency to leave. Managers should create a harmonious, healthy, and positive working environment. They should strengthen employee participation in the groups, increase the remuneration, provide career development opportunities and allow employees access support from the organization. Some scholars believe that job burnout will cause individuals to feel indifferent to their work targets and colleagues [32], and even cause turnover intention, which affects employee retention. Therefore, attention should be paid to the impact of relatives, friends, colleagues, elderly caring social organizations, and society’s on the employees mental and emotional condition. However, this study found that turnover intention III and turnover intention are generally positively correlated with social support, indicating that the higher the social support, the higher the possibility of getting other jobs and the higher turnover intention. This is contrary to the findings of a research by Duan X’s [33]. Poor mental health has been found to be common among unemployed individuals [34]. When the level of personal social support increases, the level of emotional support and material support will also increase, and the more likely it is that there will be more ways to get a new job. They succeed in their work and their strong ability to interact makes them highly likely to get other jobs. Employees of elderly caring social organizations in Anhui Province feel that their career development cannot be realized in their current institutions of work which gives them an overall willingness to leave.

### Study limitations

Some limitations in the current study should be noted. Firstly, the generalizability of our data to other employees of elderly caring social organizations in China may be limited because the sample was from one province of China only. More studies are needed to enlarge the sample selection and include more potential factors (such as other work-related factors and occupational health factors) affecting employee’s intention to leave their jobs, especially those factors for which specific interventions can be devised to reduce the turnover intention. Secondly, the causal effect could not be determined since this was a cross-sectional study,. Longitudinal studies should be considered in further studies. Finally, since we used a questionnaire in this quantitative research, there may be information bias resulting from social proof or “correctness”; however, the bias has been greatly reduced by making the participants anonymous and a well-designed questionnaire.

## Conclusions

This study provides understanding of turnover intention and its influencing factors among the Chinese employees in elderly caring social organizations. Our study showed that the turnover intention is affected by different aspects of job satisfaction and social support. Improving job satisfaction, in terms of salary, promotion, and job safety is important for reducing turnover intention. We suggest that management agencies should encourage colleagues to communicate with each other to promote mutual help, to build a good working atmosphere, enhance social support, and reduce turnover intention.

## Data Availability

The datasets used and/or analysed during the current study are available from the corresponding author on reasonable request. Anonymized participant data used in the preparation of this article will be made available on request from the lead author.

## Abbreviations

MSPSS: Multidimensional Scale of Perceived Social Support
95% CI: 95% confidence interval
S.E.: standard error
*p*: probability
r: Correlation coefficient
